# Bilateral symmetrical brachymetacarpia of the ring fingers: a case report

**DOI:** 10.11604/pamj.2025.50.108.41420

**Published:** 2025-04-23

**Authors:** Collen Sandile Nkosi, Khumoetsile Josiah Manaka, Abdulhalim Yusuf Ismail, Tatolo Ishmael Sefeane, Mmampapatla Thomas Ramokgopa

**Affiliations:** 1 Department of Orthopaedic, Chris Hani Baragwanath Academic Hospital, University of the Witwatersrand, Johannesburg, South Africa

**Keywords:** Ring finger, brachymetacarpia, brachydactyly, short metacarpals, case report

## Abstract

Brachymetacarpia is the term for the shortening of the digit as a result of the metacarpal shortening. Isolated idiopathic brachymetacarpia is rarely reported in African countries, and its incidence is still unknown in the literature compared to brachymetatarsia, which is extensively reported. We report an uncommon case of a 26-year-old male patient who presented with short, symmetrical ring fingers without an associated medical condition. Clinical examination revealed signs in keeping with brachymetacarpia, while plain radiographs showed a metacarpal sign. Non-operative treatment was given for bilateral brachymetacarpia.

## Introduction

Brachymetacarpia is characterized by digit shortening in a variety of congenital or acquired conditions, either as a solitary malformation or as a syndromic malformation [1]. It is a rare condition that affects more women than men [2]. The ring finger metacarpal is the digit that is most frequently affected and tends to occur due to premature growth plate fusion or an associated condition that affects the growth plate or bone [1,3]. The majority of cases of brachymetacarpia do not present with reduced hand function, hence, non-operative treatment is usually the standard treatment. Only a small number of patients who present with mild hand impairment and who request surgery for cosmetic reasons undergo it [4]. We report an uncommon case of a patient who presented with short, symmetrical ring fingers without an associated medical condition.

## Patient and observation

**Patient information:** a 26-year-old male presented to our emergency department after injuring himself on the left thumb with a knife while attempting to cut something at work. He had an isolated hand injury, and his right hand was dominant. He did not present any other comorbidities, and importantly, there was no history of anomalies in the family.

**Figure 1:**
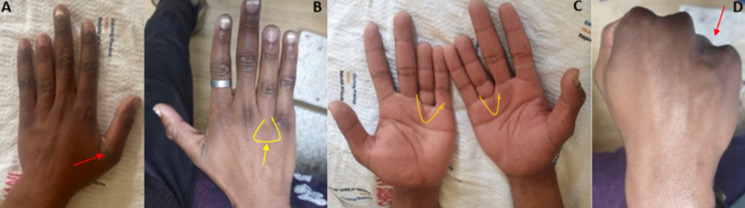
brachymetacarpia clinical signs: A) is the laceration with which the patient presented; B) the metacarpophalanges joint wrinkle sign; C) the V signs; D) the knuckle sign

**Clinical findings:** on clinical examination, he appeared to be in good health and had normal vital signs. He has a 2cm horizontal laceration on the dorsal side of his left thumb. He couldn't extend his left thumb's metacarpophalangeal joint. He had bilateral symmetrical short ring fingers with no functional limitations, except for the left thumb extensor tendon laceration. A knuckle sign on both hands was observed on clenched fists (Figure 1).

**Figure 2:**
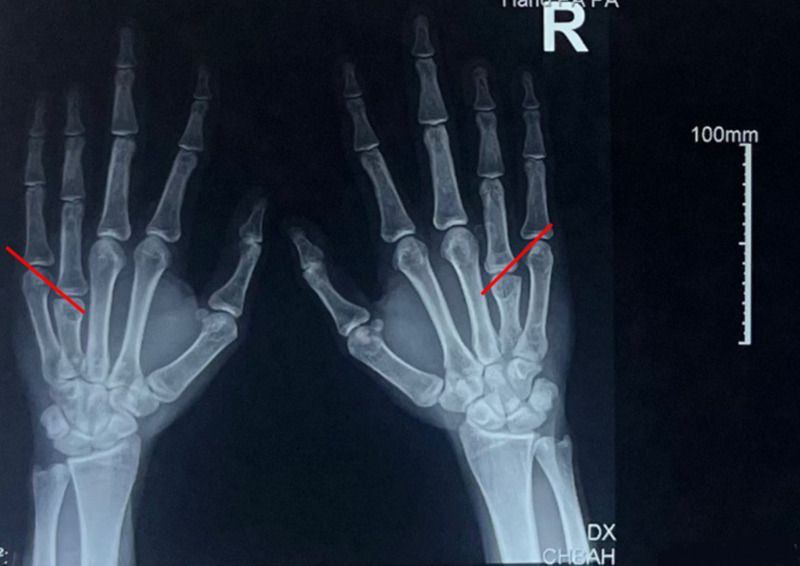
radiograph of hands showing the short 4^th^ metacarpal signs demonstrated by the red lines

**Diagnostic assessment:** a plain radiograph of the hands revealed a branchymetacarpia of the ring fingers with no associated fractures. Metacarpal signs were noted on the radiographs (Figure 2). A basic blood workup was within normal limits.

**Diagnosis:** a diagnosis of idiopathic symmetrical brachymetacarpia was made without feather investigation being performed.

**Therapeutic intervention:** the patient was admitted for the exploration and repair of the extensor tendons. Complete laceration of the extensor pollicis brevis was then repaired.

**Follow-up and outcomes:** postoperative physiotherapy for the injured thumb was started after one week and there was no intervention for the bilateral brachymetacarpia. He returned to full duties after 6 weeks.

**Patient´s perspective:** the patient was happy with operative results and non-operative management of the brachymetacarpia.

**Informed consent:** written informed consent was obtained from the patient for participation in our study.

## Discussion

Brachymetacarpia is the term for the shortening of the digit as a result of the metacarpal shortening. Isolated idiopathic brachymetacarpia is rarely reported in African countries, and its incidence is still not known to date in the literature compared to brachymetatarsia, which is extensively reported [1,4]. The fourth digit finger has been recognized as frequently being affected on both hands and feet, with no known familial aetiology [1,4]. Brachymetacarpia has been linked to a wide range of medical conditions and syndromic that affect its growth [5]. In our case, we had isolated symmetrical brachymetacarpia, categorized as type E in the Bells classification, with no known family history of genetic anomalies. Brachydactyly type E is defined as fluctuating metacarpal shortening with more or less normal phalange length [6]. Our case was assigned to a subclass of E1, considering it only affects the fourth metacarpal with normal and not linked phalanges deficiency and not variable combinations. A clinical diagnosis of brachymetacarpia is made when there is evidence of a small digit when compared to the opposite digit or the digit equal to the little finger, as in our case. A “knuckle sign” has previously been observed in patients with brachymetacarpia. When the affected digit is in the first position with the metacarpophalanges joint (MCPJ) flexed 90 degrees, the knuckle does not appear to be present [4,7]. One study demonstrated an abnormal palmar crease at the MCPJ of the ring finger in a case with metacarpia [5]. Our case had missing wrinkle MCPJ skin and V palmer crease signs, as demonstrated above.

In 1959, Archibald *et al*. described the "metacarpal sign" for the ring finger in Turner syndrome by drawing a line tangential to the 4^th^ and 5^th^ metacarpal heads that will cross over with the 3^rd^ metacarpal head, while on a normal hand, it does not [7]. This is a sign of the fourth and fifth metacarpal bones shortening, drawn on a hand radiograph [7]. This metacarpal sign has been reported in the literature as being present even in patients who do not have hereditary abnormalities [7,8]. In addition, plain radiographs would reveal a short metacarpal with a fused growth plate without or with short phalanges on the digit, and other metacarpals should be assessed for shortening [3,6]. We also advise a compulsory general examination of patients with short digits, specifically foot anomalies. Short metacarpals are often treated nonoperatively in patients unless they are symptomatic. Some centers have regarded this as the standard of treatment [1,4,5,9]. If the metacarpal arch is affected, which accounts for more than 75% of the function of the finger, brachymetacarpia may result in functional and cosmetic concerns [4,5,9]. These hand complaints include power grasp, chuck grip, key pinch, precision pinch, and a reduction in the MCPJ's range of motion is not considered in this [5,9]. Our case was treated non-operatively for the bilateral brachymetacarpia. Since hand function is usually normal, a cosmetic procedure is typically performed in more than 50% of cases. The cosmesis reason was because of knuckle signs and short-digit appearances [5,9]. Surgical intervention is advised in adolescent patients but is not necessary in adult patients [2,5,10]. Numerous lengthening procedures, such as osteotomies, intercalary bone grafting, and callotasis, have been described [2,9,10]. The complication rate currently is 36.6%, and the surgical interventions are not without concerns [2,9].

## Conclusion

A thorough history is still necessary, and a head-to-toe examination excludes any associated medical disorders. Bilateral ring fingers brachymetacarpia were diagnosed clinically with confirmation of short metacarpal on plain radiographs. The brachymetacarpia of the bilateral ring fingers in this patient was discovered incidentally, and no surgical intervention was recommended. There is no consensus on how to treat brachymetacarpia.
